# LB17. Immunosequencing of the T-Cell Receptor Repertoire Reveals Signatures Specific for Diagnosis and Characterization of Early Lyme Disease

**DOI:** 10.1093/ofid/ofab466.1653

**Published:** 2021-12-04

**Authors:** Sudeb C Dalai, Julia Greissl, Mitch Pesesky, Allison W Rebman, Mark J Soloski, Elizabeth J Horn, Jennifer N Dines, Rachel M Gittelman, Thomas M Snyder, Ryan O Emerson, Edward Meeds, Thomas Manley, Ian M Kaplan, Lance Baldo, Jonathan M Carlson, Harlan S Robins, John Aucott

**Affiliations:** 1 Adaptive Biotechnologies and Stanford University School of Medicine, Seattle, WA; 2 Microsoft Research, Redmond, Washington; 3 Adaptive Biotechnologies, Seattle, Washington; 4 Johns Hopkins University School of Medicine, Baltimore, Maryland; 5 Lyme Disease Biobank, Portland, Oregon

## Abstract

**Background:**

Changing climate and demographic trends have led to recent increases in the incidence of tick-borne illnesses. Early diagnosis of Lyme disease (LD) is critical for initiation of antibiotics to mitigate symptoms and prevent late manifestations. In patients not presenting with a typical erythema migrans rash, 2-tiered serologic testing is recommended to support a diagnosis of LD. However, 2-tiered testing is limited by ambiguity in interpretation and low sensitivity in early disease, highlighting an unmet clinical need for alternative diagnostic approaches. We identified a clinical signal for early LD based on evaluation of the T-cell response to *B. burgdorferi* infection.

**Methods:**

We immunosequenced T-cell receptor (TCR) repertoires in blood samples from 3 independent cohorts of patients with laboratory-confirmed or clinically diagnosed early LD and endemic/non-endemic controls to identify 251 public, LD-associated TCRs. These TCRs were used to train a classifier that identified early LD with 99% specificity. Classifier sensitivity was evaluated in 211 LD cases and 2631 endemic controls and compared to that of standard 2-tiered testing (STTT). Biologic specificity was assessed by correlating TCR assay scores with clinical measures and by mapping the antigen specificity of Lyme-associated TCRs to *B. burgdorferi* antigens.

Figure 1. LD-associated TCRs distinguish cases (orange) from controls (blue) in training cohorts. (A) Logistic-growth curve used to define a scoring function. (B) Positive-call threshold (99th percentile in endemic controls).

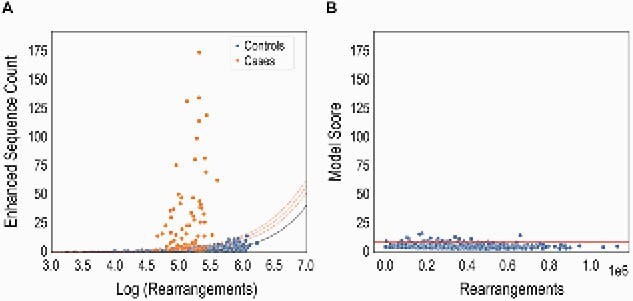

**Results:**

In early LD, TCR testing demonstrated a 1.9-fold increase in sensitivity compared to STTT (56% vs 30%), with a 3.1-fold increase ≤4 days from the onset of symptoms (44% vs 14%). TCR positivity predicted subsequent seroconversion in 37% of initially STTT-negative patients, suggesting the T-cell response is detectable before the humoral response. While positivity for both tests declined following treatment, greater declines in posttreatment sensitivity were observed for STTT compared to TCR testing. Higher TCR scores were associated with measures of disease severity, including abnormal liver function tests, disseminated rash, and number of symptoms. A subset of LD-associated TCRs mapped to *B. burgdorferi* antigens, demonstrating the high specificity of a TCR immunosequencing approach.

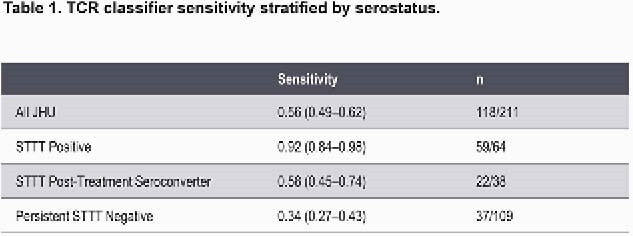

Figure 2. Validation of the TCR classifier in the JHU cohort and other holdout endemic controls. Distribution of model scores (A) and assay sensitivity (B). Model scores (C) and ROC (D) curves by serostatus.

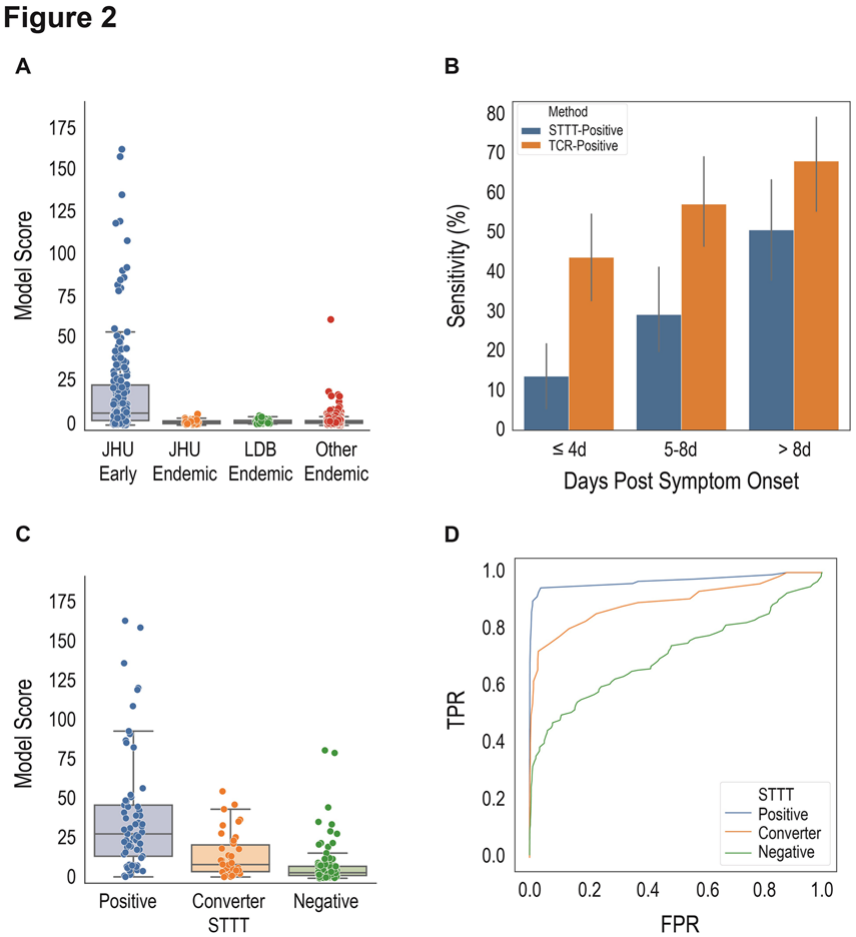

Figure 3. Clinical correlates of TCR scoring. (A) Liver function test; (B) lymphocyte count, (C) rash presentation, (D) number of symptoms.

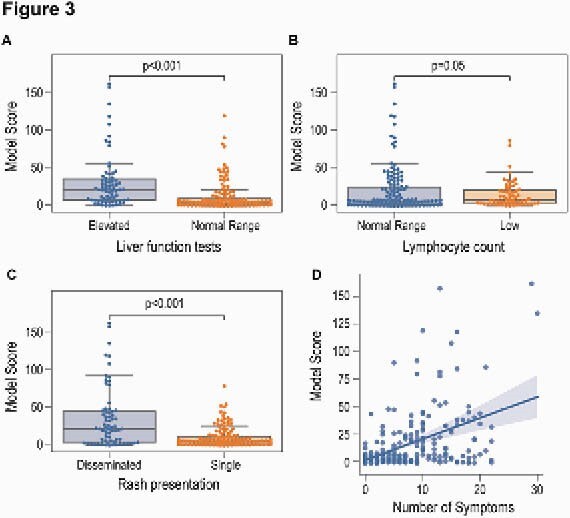

**Conclusion:**

T-cell-based testing has potential clinical utility as a sensitive and specific diagnostic for early LD, particularly in the initial days of illness.

**Disclosures:**

**Sudeb C. Dalai, MD, PhD**, **Adaptive Biotechnologies** (Employee, Shareholder) **Julia Greissl, PhD**, **Microsoft** (Employee, Shareholder) **Mitch Pesesky, PhD**, **Adaptive Biotechnologies** (Employee, Shareholder) **Allison W. Rebman, MPH**, **Global Lyme Alliance** (Research Grant or Support)**Steven and Alexandra Cohen Foundation** (Research Grant or Support) **Mark J. Soloski, PhD**, **NIH grant P30 AR070254** (Grant/Research Support)**Steven and Alexandra Cohen Foundation** (Research Grant or Support) **Elizabeth J. Horn, PhD**, **Adaptive Biotechnologies** (Research Grant or Support)**Bay Area Lyme Foundation** (Research Grant or Support)**Lyme Disease Biobank** (Employee)**Steven and Alexandra Cohen Foundation** (Research Grant or Support) **Jennifer N. Dines, MD**, **Adaptive Biotechnologies** (Employee, Shareholder) **Rachel M. Gittelman, PhD**, **Adaptive Biotechnologies** (Employee, Shareholder) **Thomas M. Snyder, PhD**, **Adaptive Biotechnologies** (Employee, Shareholder) **Ryan O. Emerson, PhD**, **Adaptive Biotechnologies** (Other Financial or Material Support, Employment with Adaptive Biotechnologies during the time of this study) **Edward Meeds, PhD**, **Microsoft** (Employee, Shareholder) **Thomas Manley, MD**, **Adaptive Biotechnologies** (Other Financial or Material Support, Declares employment with Adaptive Biotechnologies during the time of this study) **Ian M. Kaplan, PhD**, **Adaptive Biotechnologies** (Employee, Shareholder) **Lance Baldo, MD**, **Adaptive Biotechnologies** (Employee, Shareholder, Leadership Interest) **Jonathan M. Carlson, PhD**, **Microsoft** (Employee, Shareholder) **Harlan S. Robins, PhD**, **Adaptive Biotechnologies** (Board Member, Employee, Shareholder) **John Aucott, MD**, **Adaptive Biotechnologies** (Advisor or Review Panel member)**Bay Area Lyme Foundation** (Other Financial or Material Support, Scientific Advisory Board member)**Department of Health and Human Services** (Other Financial or Material Support, Past Chair, 2018, HHS Tick-borne Disease Working Group, Office of HIV/AIDS and Infectious Disease Policy, Office of the Assistant Secretary of Health)**Expert testimony** (Other Financial or Material Support, Expert testimony)**Global Lyme Alliance** (Research Grant or Support)**Pfizer** (Consultant)**Steven and Alexandra Cohen Foundation** (Research Grant or Support)**Tarsus Pharmaceuticals** (Consultant)

